# Management of chest impalement injury

**DOI:** 10.1016/j.ijscr.2019.07.043

**Published:** 2019-07-23

**Authors:** Carlo Bergaminelli, Rosario Salvi, Dario Maria Mattiacci, Giovanni Messina, Marcellino Cicalese, Carlo Curcio, Salvatore Buono, Antonio Corcione, Marco Rispoli

**Affiliations:** a Monaldi Hospital – Thoracic Surgery, Italy; b Monaldi Hospital – Anesthesia and ICU, Italy

**Keywords:** Chest wall, Trauma, Penetrating, Bleeding, Thoracic surgery

## Abstract

**Presentation of case:**

We will describe the case of a man who impaled himself on a greenhouse pole by falling off a ladder.

**Discussion:**

The belated radiological exclusion of any spine and neck lesions forced the surgeons to operate with the patient supine and on a spine board, which prevented them from performing the classic thoracotomy and reaching the entry hole in the right scapula area.

**Conclusion:**

A double thoracotomy and the expedient of a haemostatic plug, positioned simultaneously with the extraction of the pole, allowed to control bleeding with absolute safety margins.

## Highlights

• Impalement injuries are a challenging scenario involving several specialities.
• Surgeons were forced to operate in an even circumstances, not being able to turn the patient in a lateral position.
• The double thoracotomy and the expedient of the haemostatic plug allowed to control bleeding with absolute safety margins.

## 1 Introduction

An impalement injury is a challenging scenario involving several specialities. The high energy impact of these traumas creates an extensive local tissue destruction with elements of both blunt and penetrating injury. Emergency thoracotomy in thoracic trauma has significant mortality and morbidity representing 25% of trauma related mortality.

## 2 Case report

**Fig. 1:**
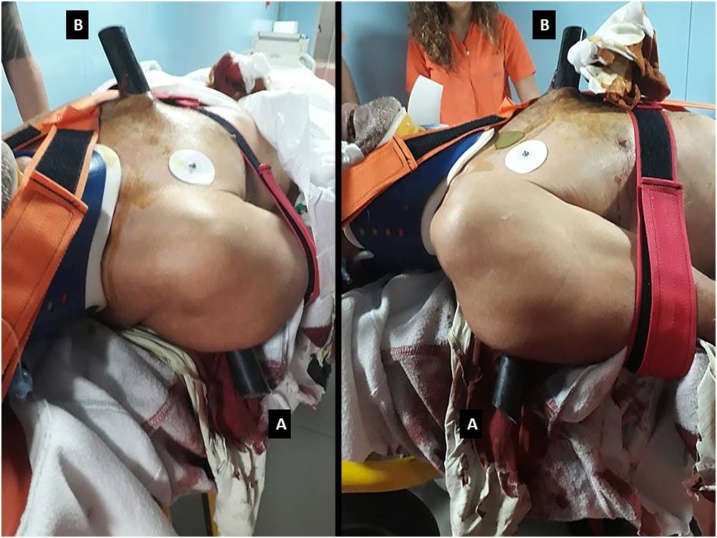
Patient impaled himself on a pole of a greenhouse. The pole had entered the right side of the back to exit the sternum. (1A) Entrance hole, (1B) exit hole.

After approval from the ethics committee, we report the case of a 61-year-old male patient (61 kg, 168 cm, BMI 22) who by falling from a ladder impaled himself on a greenhouse pole. The pole entered from the right side of the back to exit from the sternum (Fig. 1).

**Fig. 2:**
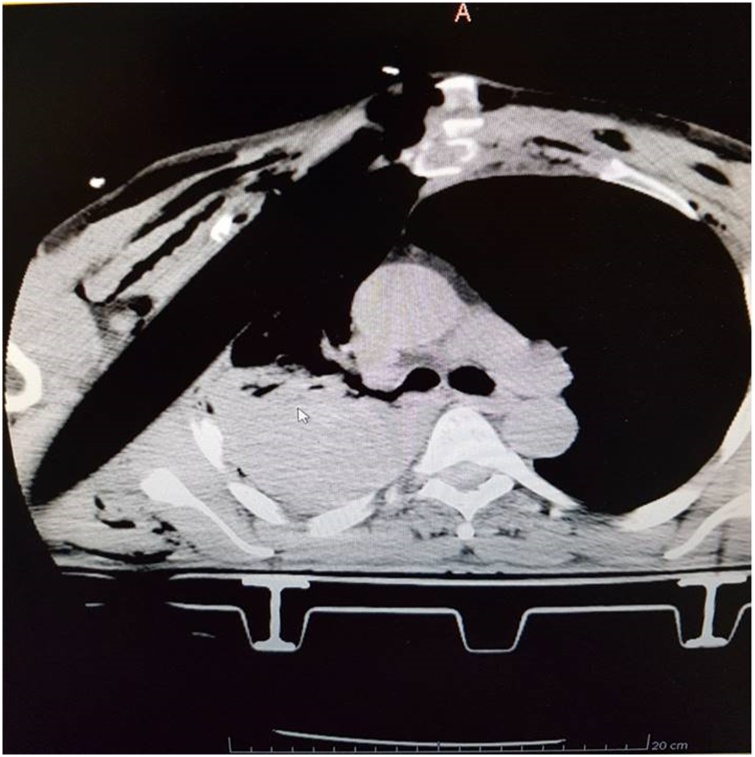
Chest CT scan: Tubuliform foreign body in the right lateral thoracic wall. Conspicuous apical-parietal-basal hemothorax with concomitant atelectasis. Front-basal right pneumothorax and decomposed fractal of the middle arch of the 3rd and 4th ribs.

After prehospital trauma care, the patient was intubated and underwent a chest CT scan reporting “Tubuliform foreign body in the right lateral thoracic wall. Conspicuous apical-parietal-basal hemothorax with concomitant atelectasis. Front-basal right pneumothorax and decomposed fractal of the middle arch of the 3rd and 4th ribs” (Fig. 2). He was then referred to the oncologic thoracic surgery unit in our hospital.

On arrival the patient underwent a secondary survey: HR 100 bpm, NiBP90/60 mmHg, SpO2 100%, T 36.2 °C. Before surgery a further radiological study was performed: CT angiography excluded any involvement of the cardiac structures and of the larger vessels of the thorax. Total body CT was also performed.

Once in the operating room, intraoperative blood recovery was set up and a rapid intravenous infuser/warmer was used for fluid management. Large chemical heat pads were applied to the lower limbs. Endotracheal tube was removed after placing a bougie and a left Double Lumen Tube was inserted.

Waiting for radiologists to exclude any injuries to the spine and upper neck region, surgery started with the patient on spine board and with a cervical collar still on, which prevented from operating on the lateral position, which is the most suited.

A right anterolateral thoracotomy at 5th intercostal space was performed. After massive hemothorax (1200 ml) was drained, the intrathoracic part of the pole was clearly displayed. Hilar pulmonary structures, pericardium and epicardial structures were uninjured. A ventral subsegmentectomy of the right upper lobe was performed with parenchymal EndoGIA^™^(Medtronic. Minneapolis, Minnesota, USA) to remove a lacerated and contused lesion. After ligation of the intercostal arteries involved, the removal of multiple sternal and costal fragments allowed the exposure of the sheared internal mammary artery and vein: haemostasis with metal clips was performed.

**Fig. 3:**
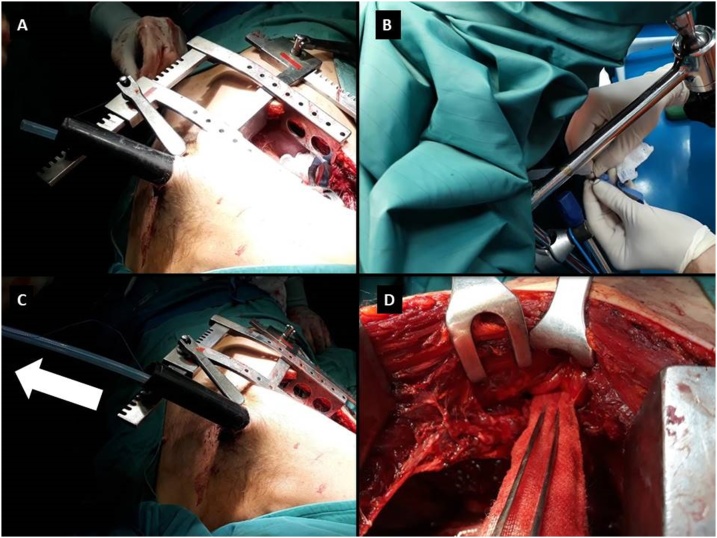
(3A) The thoracic drainage tube was passed through the hole of the pole to the outside of the operating field. (3B) A sterile gauze was fixed to the thoracic drainage. (3C) The removal of the foreign body started from the exit hole, pulling together the pole and the thoracic drainage inside of it. (3D) In this way the gauze at the end acted as haemostatic plug for the tissue injured by the pole penetration, exploiting its immediate mechanical haemostatic action.

A second right anterior thoracotomy at 3rd intercostal space, close to the thoracic intraparietal tract of the pole, was then performed to remove the foreign body. To prevent haemorrhage from the pole removal, a sterile gauze was fixed to a thoracic drainage, then the thoracic drainage tube was passed through the entrance hole of the pole (in the back) outside of the operating field. Then the removal of the foreign body started from the exit hole (in the parasternal area).In this way the gauze on one end acted as an haemostatic plug for the tissue injured by the pole penetration, exploiting its immediate mechanical haemostatic action. The pole was finally removed from the thorax (Fig. 3).

To guarantee stability and functionality of the rib cage despite the removal of the right 3rd and 4th ribs, the 2nd, 5th, and 6th right ribs were mobilized. Using a non-resorbable suture, the 2nd rib was pulled downwards and anchored to the lower ones, while the 5th and the 6th ribs were pulled upwards and anchored to the upper ones to fill the empty intercostal spaces.

Pectoralis muscle-myocutaneous flapping was performed and folded onto the contralateral pectoral fascia to cover the loss of thoracic wall. Two 28 Fr thoracic drainages were placed and a 16 Fr drainage was placed next to the entrance hole connected to a high-vacuum wound drainage systems.

After surgery the patient was referred to post-operative ICU. He was then successfully extubated. And on the 9th day a chest CT scan was performed reporting: “millimetric layer of pneumothorax, right basal pleural fluid layer, thickening band of the right upper lobe adjacent to metal suture and ground glass opacity residues from a likely outcome of alveolar haemorrhage”. On 12th day the patient underwent a spyrometric test: FVC 66% and FEV1 69% with a normal FEV1/FVC ratio as from mild restrictive pattern. Twenty days after surgery, the patient was dismissed. At one-month follow-up the patient underwent CT scan reporting no layer of pneumothorax or pleural fluid and the spyrometric test showed a mild improvement (FVC 76% and FEV1 79%). After three months a second follow-up was performed which confirmed the success of the intervention.

## 3 Comment

The patient was still awake when the emergency medical service (EMS) arrived. The impaling pole had been left in-situ but firefighters had to cut it to allow the patient to enter the ambulance. In contrast to the trauma guidelines the patient was intubated and a positive pressure ventilation started without positioning a thoracic drainage, not even with the evidence of pneumothorax at the first CT [1].

When the patient came to our observation, the haemodynamic stability and the good oxygenation profile allowed us to exclude the presence of a hypertensive pneumothorax, considering the three hours passed from the accident. The stability of the patient led us to deepen the radiological diagnosis with a CT angiography and a CT Total Body in order to investigate a possible aortic dissection or transection, which is the only scenario to defer the drainage positioning until diagnosis is obtained [2].

The need for double anterior thoracotomy depended, on one hand, on the belated exclusion of injuries to the spine and upper neck region by radiologists which prevented the patient’s lateral positioning and, on the other, on the necessity to inspect any bleeding in progress and to prepare the tissues for extraction of the foreign body from an access different from the exit hole of the pole. Likewise, the lack of information about neck and spine stability forced surgeons to secure effective haemostasis by using the gauge as haemostatic plug while removing the foreign body. In fact any bleeding from soft tissue in the scapula area would have been difficult to stop considering the spine board and the supine position.

Rigid chest-wall reconstruction in the presence of surgical-site infection can result in significant morbidity and mortality, ranging from 9% to 47% [[3], [4], [5]]. Therefore, the costal mobilization and pectoralis muscle-myocutaneous flapping onto the contralateral pectoral fascia to cover the loss of thoracic wall was performed to avoid prosthetic reconstruction of the chest wall, taking into account the potential risk of infection in a patient with penetrating trauma in a rural environment despite ongoing antibiotic therapy.

Impalement injuries are a challenging scenario involving several specialities. In this case the delay in radiological exclusion of any spine and neck lesions forced the surgeons to operate in an uneven circumstances, not being able to turn the patient onto a lateral position. The double thoracotomy and the expedient of the haemostatic plug positioned simultaneously with the extraction of the pole allowed to control bleeding with absolute safety margins.

## Sources of funding

Authors did not receive funding for this research.

## Ethical approval

Patient gave us informed consent and ethnical approval was not required.

## Consent

Written informed consent was obtained from the patient for publication of this case report and accompanying images. A copy of the written consent is available for review by the Editor-in-Chief of this journal on request. No alteration of scientific meaning derive from identifying data omission.

## Registration of research studies

This paper is not a research on human participants.

## Guarantor

Carlo Bergaminelli.

## Provenance and peer review

Not commissioned, externally peer-reviewed.

## CRediT authorship contribution statement

**Carlo Bergaminelli:** Conceptualization, Investigation, Writing - original draft. **Rosario Salvi:** Methodology, Data curation. **Dario Maria Mattiacci:** Conceptualization, Data curation, Writing - review & editing, Visualization, Supervision. **Giovanni Messina:** Validation, Writing - review & editing, Visualization. **Marcellino Cicalese:** Validation, Data curation. **Carlo Curcio:** Methodology, Writing - review & editing. **Salvatore Buono:** Methodology, Writing - original draft. **Antonio Corcione:** . **Marco Rispoli:** Conceptualization, Investigation, Writing - original draft, Writing - review & editing, Supervision.

## Declaration of Competing Interest

Authors have no conflict of interest.
